# The logic model for Uganda’s health sector preparedness for public health threats and emergencies

**DOI:** 10.1080/16549716.2019.1664103

**Published:** 2019-09-17

**Authors:** Alex Riolexus Ario, Issa Makumbi, Lilian Bulage, Simon Kyazze, Joshua Kayiwa, Milton Makoba Wetaka, Juliet Namugga Kasule, Felix Ocom

**Affiliations:** a Ministry of Health of Uganda, Kampala, Uganda; b Uganda Public Health Fellowship Program, Ministry of Health, Kampala, Kampala, Uganda; c Uganda National Institute of Public Health, Kampala, Uganda; d Public Health Emergency Operations Program, Kampala, Uganda; e African Field Epidemiology Network, Kampala, Uganda; f Public Health Emergency Operations Centre, Kampala, Uganda; g US Centers for Disease Control and Prevention, Kampala, Uganda; h WHO Country Office, Kampala, Uganda

**Keywords:** Emergencies, multi-hazard, logic model, preparedness, Uganda

## Abstract

**Background**: Uganda is an ecological hot-spot with infectious disease transmission belts which exacerbates its vulnerability to epidemics. Its proximity to the Congo Basin, climate change pressure on eco-systems, increased international travel and globalization, and influx of refugees due to porous borders, has compounded the problem. Public Health Events are a major challenge in the region with significant impact on Global Health Security.

**Objective**: The country developed a multi-hazard plan with the purpose of harmonizing processes and guiding stakeholders on strengthening emergency preparedness and response.

**Method**: Comprehensive risk profiling, identification of preparedness gaps and capacities were developed using a preparedness logic model, which is a step by step process. A multidisciplinary team was constituted; the Strategic Tool for Analysis of Risks was used for risk profiling and identification of hazards; a desk review of relevant documents informed the process and finally, approval was sought from the National Task Force for public health emergencies.

**Results**: Target users and key public health preparedness and response functions of the multi-hazard plan were identified. The key capabilities identified were: coordination; epidemiology and surveillance; laboratory; risk communication and social mobilization. In each of these capabilities, key players were identified. Risk profiling classified road traffic accident, cholera, malaria and typhoid as very high risk. Meningitis, VHF, drought, industrial accidents, terrorism, floods and landslides were high risk. Hepatitis E, avian influenza and measles were low risk and the only plague fell into the category of very low risk. Risk profiling using STAR yielded good results. All risk categories required additional preparedness activities, and very high and high-risk categories required improved operational response capacity and risk mitigation measures.

**Conclusion**: Uganda successfully developed a national multi-hazard emergency preparedness and response plan using the preparedness logic model. The plan is now ready for implementation by the Uganda MoH and partners.

## Background

Uganda is plagued with a high burden of communicable and non-communicable diseases (NCDs) resulting in poor health indicators in the country. Seventy five percent of the diseases are preventable and amenable to public health interventions. Communicable diseases contribute over 50% of disability adjusted life years lost [1]. Major health outcome indicators are still below targets, for example, under-five mortality rate is 53 deaths per 1,000 live births, infant mortality rate stands at 43 per 1000 live births and stunting in children under-five is estimated at 29% [2]. The HIV prevalence is still high at 6.2% [3] and TB prevalence is 303/100000 with emergence of MDR-TB in many parts of the country [4]. The growing burden of NCDs with inadequate focus or strategy to address them is a worrying trend [5].

In the recent past, the country has had technological, man-made and natural disasters including terrorist attacks, disease outbreaks, famines, landslides and flooding with resultant huge human and economic costs. Public Health Events (PHE) including emerging and re-emerging infectious diseases are a major challenge to Uganda and the Great Lakes region with significant impact on Global Health Security [6].

Uganda is an ecological hot spot with several infectious disease transmission belts which exacerbates its vulnerability to epidemics. Its proximity to the biodiversity rich ‘hot spot’ Congo basin compounds the problem further. The country has also experienced climate change pressure on ecosystems and environment. There is increased international travel and globalization, in addition to porous borders. There has been increased population movements in the region due to armed conflict in neighboring countries such as the Democratic Republic of Congo, Somalia, Burundi and South Sudan, with Uganda bearing the brunt of the end destination for these refugee migrations. This influx of refugees comes with many ramifications in their areas of reception and settlement in the destination country including serious public health challenges due to lack of essential commodities, food scarcity, disease outbreaks and inadequate health services [7,8].

Due to its open policy of refugee entry and settlement, Uganda has in turn experienced numerous public health threats and events that have tested her preparedness to prevent, detect and mount a timely and appropriate response aimed at saving lives and mitigating socio-economic damage and livelihood disruptions.

In 2010, the Office of the Prime Minister developed a National Policy for Disaster Preparedness and Management aimed at guiding a coordinated response to all hazards in the country. As a follow-up, the Ministry of Health (MoH) commissioned a Country Health Disaster Risk Management Capacity Assessment, which recommended among others the development of a national disaster preparedness and management plan [9].

The country developed the National Multi-hazard Emergency Preparedness and Response Plan (NMEPRP) with the purpose of harmonizing processes and guiding stakeholders on strengthening emergency preparedness and response. The plan focused on addressing health risks associated with several hazards of great magnitude. The specific objectives of the NMEPRP are to: provide a mechanism for assessing and identifying risks and hazards that pose the greatest threat to health and property in Uganda using scientific and reliable methods; prevent and reduce the likelihood of disease outbreaks and other public health hazards; to build the country’s capacity and capability to detect public health threats early; and guide a coordinated, rapid, effective and multi-sectoral response to public health threats and emergencies [10]. The NMEPRP is a guiding document which is applicable in different emergency situations that require public health response. It is a living document that will be regularly reviewed and updated to address changes in hazards, risk profiles and scenarios. It is envisaged that the plan will contribute to strengthening emergency preparedness, building resilience and initiating timely and adequate response at national and subnational levels.

Uganda’s preparedness and response to public health threats are a shared responsibility that can only be achieved through a 'whole of government’ approach. Adoption of the all-hazards response model as the fundamental paradigm provides powerful improvements in efficiency and efficacy, because it reduces the need to create situation-specific preparedness and response activities [11]. For instance, Uganda has Standard Operating Procedures (SOPs) for all emergency preparedness and response activities, regularly conducts training, drills and simulation exercises with multidisciplinary teams to test its ability to respond appropriately. The NMEPRP’s implementation success therefore hinges on collaboration and stakeholder involvement of the health, security, environment, veterinary, agriculture, non-governmental organizations and community-based organizations concerned with implementation of International Health Regulations (IHR) using the One Health approach. In this paper, we document the process of developing the plan and outline the salient health sector preparedness and response capacities/functions.

## Methods

A preparedness logic model is the method that Uganda used to perform comprehensive risk profiling, identify preparedness gaps and determine response capacities that would constitute the NMEPRP. The logic model used was adapted from the original version which was created to capture elements of the US preparedness enterprise using four approaches [12]. This is a step-by-step process logic model which has been tested and proven to produce successful multi-sectoral plans. First, we constituted a multidisciplinary team of Subject Matter Experts (SMEs) in technical areas which are responsible for preparedness and response to hazards. Some of these SMEs are members of the National Task Force (NTF) on epidemic preparedness and response, while others are from Non-Governmental Organizations, Implementing Partners, Development Partners, relevant ministries, departments and agencies of government. The approaches used during the discussions included: brainstorming, group discussions, plenary presentations and consensus buildings. The second process was adaptation of the World Health Organization (WHO) Strategic Tool for Analysis of Risks (STAR) [13]. This tool was chosen because it provides for integrated multi-hazard and multi-sectoral public health risk assessments which include exposure, vulnerability and capacity analyses. The third process was a desk review of relevant documents from the Government of Uganda, World Health Organisation (WHO), United Nations Office for Disaster Risk Reduction and other countries. Many of the documents reviewed were international and national guidelines, policies and legal frameworks on public health emergencies (Table 1). The fourth process was presentation of the NMEPRP to the National Task Force for public health emergencies (NTF) for approval. The NTF is a multidisciplinary and multi-sectoral committee which provides oversight and makes decisions which guides epidemic preparedness and response in the country. The membership includes different government line ministries, departments and agencies; international agencies, bilateral agencies, Non-Governmental Organisations and other relevant actors.

**Table 1.** Logic model for public health threats and emergencies preparedness and response in Uganda.

**Program**: National Multi-hazard Emergency Preparedness and Response Plan development		
**Goal**: Harmonizing processes and guiding stakeholders on strengthening emergency preparedness and response		
INPUTS	ACTIVITIES	OUTCOMES
What we invested	What we did	Why did we do this
SMEs on: Coordination Epidemiology Surveillance Laboratory Risk Communication Social Mobilization Other capabilities Funds	Constituted a team of multidisciplinary SMEs Adaptation of WHOs Strategic Tool for Analysis of Risks (STAR) Review of relevant documents Presentation of the plan to the NTF	Addressing health risks associated with several hazards of great magnitude Providing a mechanism for assessing and identifying risks and hazards that pose the greatest threat to health Preventing and reducing disease outbreaks and other public health hazards Building the country’s capacity and capability to detect public health threats early Guiding a coordinated, rapid, effective, and multi-sectoral response to public health threats and emergencies Strengthening emergency preparedness Building health system resilience Initiating timely and adequate response at national and subnational levels
**Assumptions** Partners will fund preparedness and response Community uptake will be adequate	**External Factors** Positive and negative influences Culture, economics, politics, demographics	

## Results

Development of the plan took into consideration objectives of epidemic preparedness and response which include: prediction so that epidemics can be prevented; detected early; rapidly and effectively responded to; and availability of resources such as guidelines/trained staff/SOPs/supplies before epidemics occur. Using the preparedness logic model, the target users and key public health preparedness and response functions of the NMEPRP were identified (Table 2). In addition, risk profiling and mapping were conducted. In the model, all aspects of resources, activities, outputs and outcomes were considered. The various categories of resources included were: personnel, funding, logistics and supplies. Major activities, implementation mechanisms and the desired outputs and outcomes were outlined throughout the different stages of the model.

**Table 2.** Categorising risk and preparedness actions required.

	Risk				
Public Health Event	Likelihood	Impact	Score	Level of risk	Preparedness action required
Road traffic accidents	5	5	25		Additional preparedness is required for this category of PHE with enhanced operational response capacity and risk mitigation measures
Cholera	5	4	20	Very high	
Malaria	5	4	20		
Typhoid fever	5	4	20		
Meningitis	4	4	16		Additional preparedness is required for this category of PHE with enhanced operational response capacity and risk mitigation measures
VHF	4	4	16		
Drought	3	5	15		
Industrial accidents	3	4	12	High	
Terrorism	3	4	12		
Floods	3	4	12		
Landslides	3	4	12		
Hepatitis E	3	3	9		Additional preparedness is required for this category of PHE but without enhanced operational response capacity and risk mitigation measures
Measles	5	2	10	Moderate	
Avian influenza	2	2	4		
RVF	2	2	4	Low	Additional preparedness without enhanced mitigation measures
Zika	2	2	4		
Plague	2	1	2	Very low	Additional preparedness

### Target users

The broad range of target users identified were health leaders, health workers, emergency responders, policy makers, line ministries, departments, donors, non-governmental organisations, private sector and other relevant agencies which contribute directly or indirectly to emergency preparedness, resilience and response of communities at risk.

### Key public health preparedness and response components of the NMEPRP

**Figure 1.**
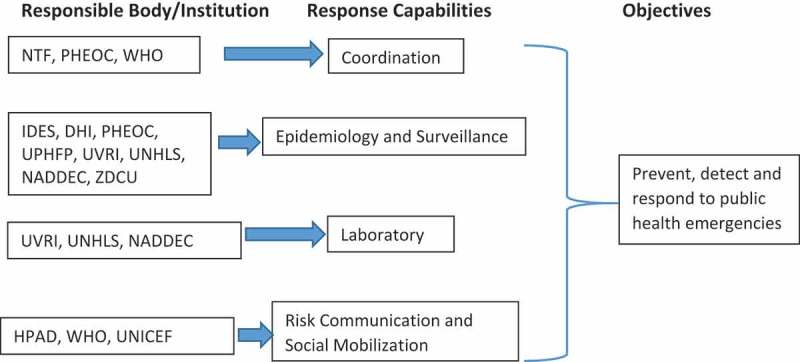
Response capabilities for public health threats preparedness in Uganda. Adapted from Michael A. Stoto et al: Health Security Volume 15, Number 5, 2017, Mary Ann Liebert, Inc. DOI: 10.1089/hs.2016.0126

The key capabilities identified include coordination; epidemiology and surveillance; laboratory; risk communication, response capabilities and social mobilization (Figure 1).

**a) Coordination**

The NTF, a structure under the MoH created to coordinate emergency public health response and chaired by the Director General of Health Services (DGHS) and co-chaired by Director Animal Resources in the Ministry of Agriculture, Animal Industry and Fisheries, is responsible for coordination of government, development partners and other relevant stakeholders who may wish to get involved in the response activities. The NTF works through technical sub-committees which carry out the day to day duties of emergency preparedness and response based on their terms of reference.

The Public Health Emergency Operations Centre (PHEOC) under MoH is responsible for coordinating information and resources (human and physical), organizing, conducting and managing all aspects of public health emergency response efforts of the country. The PHEOC is activated by the DGHS and the level of activation is dependent on the gravity and magnitude of the public health event.

**a) Epidemiology and Surveillance**

The department of Integrated Disease Epidemiology and Surveillance (IDES), Division of Health Information (DHI), PHEOC, Uganda Public Health Fellowship Program (UPHFP) and the National Reference Laboratories [Uganda Virus Research Institute (UVRI), Uganda National Health Laboratory Services (UNHLS)] under MoH are responsible for disease surveillance in the country. There are mechanisms that these departments use to link with sub-national levels for effective response. In addition, the National Animal Disease Diagnostics and Epidemiology Centre (NADDEC) under Ministry of Agriculture, Animal Industry and Fisheries (MAAIF) and the Zoonotic Disease Coordination Unit (ZDCU) helps in surveillance of zoonotic diseases.

The National Rapid Response Team (NRRT) and District Rapid Response Teams (DRRTs) are responsible for responding to PHEs. In addition, the UPHFP spearheads the epidemiologic investigations during public health emergencies.

**b) Laboratory**

Specimens from suspected case patients (humans or animals) are sent for testing and confirmation to regional referral hospitals or national reference laboratories (UNHLS, UVRI and NADDEC) through the national specimen transport and referral network also known as the hub system.

**c) Risk Communication and Social Mobilization**

The Health Promotion and Advocacy Division of MoH together with partners involved in communication spearhead the risk communication and social mobilization component of the response.

### Risk profiling in Uganda

Using the STAR, we conducted the risk profiling and mapping and classified them as very high risk, high risk, medium risk, low risk and very low risk. All categories were found to require minimal and additional preparedness activities. In addition, the very high and high-risk category required operational response capacity and risk mitigation measures. Table 2 summarizes the risk profiling for Uganda.

## Discussion

The process documentation presented in this article, with its associated adaptation of a proven logic model, provides an opportunity to show outcome of such approaches in planning for public health emergency preparedness, response and other related undertakings. Health leaders, health workers, emergency responders, policy makers, line ministries, departments, donors, non-governmental organisations and other agencies which contribute directly or indirectly to emergency preparedness, resilience and response of communities at risk were identified as target users of the NMEPRP. Salient components for health sector emergency preparedness identified were: coordination; epidemiology and surveillance; laboratory; risk communication and social mobilization. Risk profiling and mapping classified road traffic accidents, cholera, malaria and typhoid as very high risk, and meningitis, VHF, drought, industrial accidents, terrorism, floods and landslides as high risk.

Following the logic model, preparedness planning took into account contribution of subject matter experts and various partners. This produced good results as corroborated by Stoto et al. in a paper on Assessing Preparedness for Cross-border Threats in the European Region where they adapted the US preparedness logic model [14]. Another paper on disaster and emergency planning by Alexander et al. states that preparedness planning involves a coordinated, co-operative process of preparing to match urgent needs with available resources [15]. Emergency planning therefore requires employing processes that enable harnessing procedures that bring unforeseen impacts in context and craft scenarios able to anticipate the needs that will be generated by foreseeable hazards when they occur.

Risk profiling using the STAR yielded good results. This tool provides an opportunity to conduct a multi-hazard and multi-sectoral public health risk assessment with comprehensive capacity and vulnerability analyses. The tool enables integration of evidence-based approach to risk assessment in a comparable, reproducible and defensible manner. Also, helps guide thinking and group analysis as well as facilitate data entry on threats and identified public health risks; enables prioritization of major hazards in the country; and provides evidence of potential needs for strengthening national capacities in emergency preparedness and response. The tool has been used by many countries with relative success. In 2017, South Sudan became the 20^th^ African country to use STAR. South Sudan has historically been plagued by natural and man-made hazards, majority being biological hazards like the Ebola outbreaks of 1976, 1979 and 2004, Yellow fever, Cholera, Measles, Meningitis and others, perennial floods, occasional droughts and famine affecting both human and animal health. In order to effectively mitigate, reduce the impact and enhance emergency preparedness and response, South Sudan conducted a multi-sectoral risk profiling exercise using STAR to guide discussions, reflections and analysis, as well as to facilitate the capture of data on the public health threats and risks identified. After a critical analysis, the high priority public health threats were identified [16].

Profiling of hazards enabled categorization into very high, high, moderate, low and very low risks. In Uganda, the very high-risk hazards included road traffic accidents, cholera, malaria and typhoid, while meningitis, VHF, drought, industrial accidents, terrorism, floods and landslides were classified as high risk. This does not differ very much from South Sudan and Ghana risk profiles which were done using the same tool. In South Sudan, in a risk profiling exercise conducted in 2017, injuries and cholera, Malaria, Measles ranked in the category of very high risk and Hepatitis E, VHF, gender-based violence and psychological trauma occupied the high-risk category (3). While in Ghana, the top hazards were EVD, road traffic accidents, pandemic influenza, meningitis and cholera; and high-risk category had floods, terrorism, avian influenza, yellow fever [17]. There are glaring similarities observed amongst these African countries. All categories of risks will require additional preparedness activities. This implies that the capacity to meet the IHR regulations is low for these categories of PHEs. Some of the additional preparedness activities include: identifying, mobilizing and prepositioning stockpiles; building surge capacity (public health work force, logistics, partners, private) to respond; maintaining structurally and functionally safe health facilities; testing and updating capabilities at national, and district levels through either real-life situations or simulation exercises. It is important, however, to note that hazards in very high and high-risk categories will require further additional enhanced operational response capacity and risk mitigation measures.

The key capacities identified are in line with the WHO’s Strategic Framework for Emergency Preparedness which guides countries in adopting the major lessons of previous initiatives and determination of their priorities to enable them identify elements of effective country health preparedness and strengthen their operational capacities [18].

Coordination is one of the key preparedness for public health threats and emergencies capacities identified during the multi-hazard planning process. The NTF which brings on board all stakeholders is the overall coordinating unit. A functional NTF is critical in implementation of emergency preparedness plans. This has been made possible by formation of subcommittees in areas which address core preparedness and response functions such as coordination, surveillance and laboratory, case management and infection prevention and control, logistics and psychosocial support. The cascading of the structure to sub-national levels as a District Task Force ensures nation-wide reach on implementation of the preparedness and response plan. On behalf of NTF, the chair declares an outbreak and declares the end of an outbreak. The NTF is scheduled to meet quarterly when there is no emergency but as often as necessary (often twice a week or daily) if required during an emergency. The International Health Regulations (2005) requires that member state parties should have a focal point who should be a national centre for urgent communications under the IHR (2005). Since then, Uganda has a designated IHR Focal Point that is a member of the NTF and is accessible at all times and communicates with WHO concerning consultations, notifications, verification and assessments of public health events.

The PHEOC is responsible for coordinating information and resources involved in managing all aspects of public health emergency response effort of the country. The PHEOC monitors public health events both at national and international levels using event-based surveillance and Indicator-based surveillance, and follows up alerts; then informs relevant sectors for action.

Effective preparedness and response rely on monitoring disease patterns, investigating individual case reports, and using epidemiological and laboratory analyses to target public health intervention strategies. Prompt recognition and reporting of cases to health authorities is a critical link in the public health chain. Public health surveillance is performed using the Integrated Disease Surveillance and Response (IDSR); however, in some instances, specific surveillance systems are set up as necessary for more effective surveillance [19].

There are Standard Operating Procedures which guide the operations of the NRRT and DRRT. The information they collect from the field investigations helps NTF take decisions which streamline response to public health emergencies.

In many situations, laboratories provide the definitive identification of causative agents, both biological and chemical, and through various fingerprinting activities link cases to a common source. Capabilities to identify rare or unusual diseases is not available at lower level laboratories hence, necessitating linkages with higher level laboratories. Specimens may be sent for testing and confirmation to a regional referral hospital laboratory or national reference laboratories (UNHLS, UVRI and NADDEC).

Effective communication is key in the management of emergencies and development of resilience to prevent and/or mitigate the impact of disasters. Uganda has a risk communication strategy and IEC materials on major health problems that has been translated into major local languages. Management of and collaboration with the media is recommended.

Road traffic accidents, cholera, malaria and typhoid were classified as very high risk, while meningitis, VHF, drought, industrial accidents, terrorism, floods and landslides were classified as high risk; Hepatitis E, avian influenza and measles were classified as low risk and only plague fell into the category of very low risk. Though all categories will require minimal and additional preparedness activities, the very high and high-risk category will require operational response capacity and risk mitigation measures.

## Conclusion

Uganda was able to successfully develop a national multi-hazard emergency preparedness and response plan using the preparedness logic model. The plan is now ready for implementation by the Uganda Ministry of Health and its partners.

## Paper context

Uganda has been able to successfully develop a national multi-hazard emergency preparedness and response plan using the preparedness logic model. Using the STAR tool, risks were categorized.
